# Preventing lower limb injuries in women’s football: Influence of the *Stop X* prevention programme on jump performance and isokinetics in professional and young elite-level female football – a prospective observational cohort study

**DOI:** 10.1186/s13102-026-02064-2

**Published:** 2026-09-09

**Authors:** Jonas Seiler, Laura Keller, Matthias Rosa, Dominik Gnädinger, Gerrit Bode, Hagen Schmal, Lisa Bode

**Affiliations:** 1 Department of Orthopaedic Surgery and Traumatology, Freiburg University Hospital, Albert Ludwigs University Freiburg, Freiburg, Germany; 2 SC Freiburg e.V, Freiburg, Germany; 3 Sportklinik Freiburg, Freiburg, Germany; 4 Department of Orthopaedic Surgery, University Hospital Odense, Odense, Denmark

**Keywords:** Women’s football, Non-contact injury prevention, Neuromuscular training, Isokinetic strength, Jump performance, Stop X

## Abstract

**Background:**

Lower limb injuries, particularly anterior cruciate ligament (ACL) and thigh injuries, are highly prevalent in women’s football, often resulting from sudden changes in direction and poor landing mechanics. Neuromuscular training is critical to address these injury risk factors. This study evaluated the adaptions observed during implementation of the Stop X programme, a specialised neuromuscular training developed by the German Knee Society, on jump performance and isokinetic strength in elite female players.

**Methods:**

In this prospective observational cohort study, 147 female players (Professional: *n* = 55, U20: *n* = 34, U17: *n* = 58) were monitored during 2022–2024 seasons. *Stop X* was integrated into the team warm-up (5–6 sessions/week, 15–20 min). Pre-season and in-season assessments included countermovement jump (CMJ), single-leg drop jump (SLDJ), and isokinetic strength testing (60°/s). Non-contact injuries were documented by the medical staff. Statistical analysis compared longitudinal performance changes and baseline values between injured and uninjured players.

**Results:**

Significant inseason improvements were observed in CMJ and SLDJ performance, with youth elite players (U17/U20) showing the most pronounced gains in reactive strength index (RSI) and jump height. In the U20 group, non-dominant leg propulsive force increased significantly (*p* = 0.04). Regarding isokinetic strength, tests revealed a significant inseason decline in the functional H/Q ratio (60°/s) for both the dominant (preseason: 0.79 ± 0.15 vs. inseason: 0.75 ± 0.13; *p* = 0.004) and non-dominant leg (preseason: 0.80 ± 0.14 vs. inseason: 0.76 ± 0.14; *p* = 0.005), while the conventional H/Q ratio showed no significant changes. Furthermore, matched non-contact injured players exhibited significantly lower in-season conventional H/Q ratios in the dominant leg compared to uninjured players (0.54 vs. 0.59, *p* = 0.03). ACL-injured players also showed a trend toward increased knee valgus in pre-injury tests (*p* = 0.06).

**Conclusion:**

Improvements in jump performance and propulsive forces were observed during the implementation of *Stop X*, providing a crucial performance-based incentive for coach and player compliance. However, the significant in-season decline in functional H/Q ratios indicates the need to refine prevention programmes by integrating more female-centric hamstring exercises alongside jumping and landing technique drills. During the implementation period, a low overall number of injuries was recorded; future randomised controlled trials with precise exposure tracking are warranted to investigate definitive injury reduction rates.

**Supplementary Information:**

The online version contains supplementary material available at https://doi.org/10.1186/s13102-026-02064-2.

## Background

A high prevalence of lower limb injuries, including those affecting the knee, ankle, hip, and hamstring, has been documented among female football players [1]. Consistent findings from epidemiological studies indicate that there are significant sex-based differences in injury patterns. Women are more likely to sustain anterior cruciate ligament (ACL), knee, and ankle injuries than men [2, 3]. In addition to the biomechanical aspects of injuries, female football players may experience an elevated injury risk due to a combination of anatomical, hormonal, and neuromuscular factors [3, 4]. Data suggest that in a typical women’s squad of 23 players, there is a 70% chance of at least one ACL injury per season [5]. Furthermore, women are more than twice as likely as men to suffer an ACL injury, and they tend to sustain these injuries at a younger age [6]. Additionally, ACL injuries are one of the most burdensome injuries [6]. The long-term impact is significant—only about 65% of professional female athletes regain their previous performance level within three years after an ACL injury [7]. Other common and impactful ligament injuries include lateral ankle sprains and medial collateral ligament injuries, both of which can also lead to extended absences [5, 8].

Biomechanical analyses indicate that ACL injuries often result from sudden directional changes, jump landings, or non-contact twisting movements that stress the knee and ankle joints [9, 10]. Approximately 70% of knee injuries in female football players occur in non-contact situations, highlighting the importance of targeting these types of injuries in prevention strategies [11, 12]. Several injury prevention programmes have been developed focusing on reducing non-contact ACL, knee, and ankle injuries [12]. Evidence suggests that multicomponent exercise-based programmes may have a beneficial effect on the prevalence of both overall injuries and ACL injuries in female football players, with a reported reduction of 27% and 45%, respectively [9, 13]. However, the existing literature still lacks comprehensive data focused on adolescent and adult female football players, in particular young elite-level female footballers.

To address this gap, in the present study we investigated the *Stop X* neuromuscular training programme developed by the German Knee Society to correct risky movement patterns and strengthen muscles critical for knee stability [14]. The name “*Stop X*” reflects its goal of reducing excessive dynamic valgus (“X” position of the knees), a primary risk factor for ACL injuries. However, there is a lack of empirical data on the efficacy of the *Stop X* programme in preventing injuries in elite adolescent and adult female football players, particularly regarding its impact on jumping ability and isokinetic testing. For instance, studies have demonstrated that jump tests serve to identify surrogate markers for non-contact cruciate ligament injuries, including dynamic knee valgus, particularly in female athletes [15].

Therefore, the aim of this study is to evaluate the influence of the *Stop X* prevention programme on injury surrogate factors by analysing changes in jumping performance and isokinetic test outcomes in female football players.

## Methods

### Study design, participants and monitoring of injuries

This study constitutes a prospective observational cohort study designed to investigate the neuromuscular and biomechanical adaptations associated with the implementation of the Stop X prevention programme. The primary outcomes and a priori hypotheses of this study were: (1) an improvement in injury-related surrogate markers, specifically the H/Q ratio and frontal plane knee kinematics (knee valgus) at initial contact and (2) an improvement in neuromuscular jump performance (e.g., the Reactive Strength Index (RSI)). Secondary outcomes included additional jump characteristics, such as Net Peak Propulsive Force (PPF) and Jump Height. Furthermore, an exploratory risk analysis was conducted to investigate potential associations between the collected jump and isokinetic data (pre-season and mid-season) and the injury data recorded during the season. This analysis aimed to identify descriptive trends in the biomechanical profiles of injured versus non-injured players, acknowledging the limited statistical power due to the small number of injury events.

Our study athletes are female footballers playing for a German professional football club. All Players from the first team competing in the 1st Women’s national league (Bundesliga); the U20 team competing in the youth third national league (Regionalliga); and the U17 team competing in the youth first national league (Bundesliga Süd) were prospectively included in our study (see inclusion and exclusion criteria in Table 1). This resulted in a total of 77 players for that season. We also included these teams retrospectively for the 2022/23 season, encompassing thereby a total of 70 players. As the training programme was carried out by the entire teams as part of their training sessions, there was no randomisation and no control group of players.

**Table 1:** Inclusion and exclusion criteria of study participants

Inclusion Criteria	Exclusion criteria
Female football players playing for a German Professional football club	Acute injury or illness at the time of measurements
Age 14–35 (U17: under 17y, U20: 17-20y, Professional: >20 y)	Players with chronic injuries (e.g. pubitis)
Continuous participation in team training between measurements for at least 8 weeks	

During the 2023/24 season, a weekly survey of all teams was conducted (assisted by physiotherapists) to monitor injuries. Data were collected using a standardised injury surveillance form (see Supplementary Material S1) and were subsequently transferred into a standardised electronic database (Microsoft Excel) for further analysis and team-specific monitoring. This form captured comprehensive details for each incident. The documentation was performed by the team’s medical staff to ensure clinical accuracy. Injuries to the lower extremity that occurred in a non-contact situation and resulted in at least two weeks’ absence were analysed. Injuries that occurred in other parts of the body (e.g. the hand), occurred in contact situations, or resulted in downtime of less than 14 days were excluded. Injuries sustained during the 2022/23 season were analysed retrospectively by assessing the players’ medical records. Players with a history of lower limb injuries were not excluded but the injury history was documented and accounted for during the matching process. While team and player adherence to the training sessions was monitored, precise individual exposure time (i.e., exact minutes of total training and match play) was not recorded. Consequently, true injury incidence rates (e.g., injuries per 1000 h of exposure) could not be calculated. The exploratory analysis therefore relies on absolute injury counts.

This study was approved by the Ethics Committee of the Albert Ludwig University of Freiburg (Reference number: 86/20) and was conducted in accordance with the Declaration of Helsinki. Written informed consent was obtained from all participants prior to the study; for participants under the age of 18, written consent was obtained from their legal guardians. The study was registered with the German Clinical Trials Register (Deutsches Register Klinischer Studien; Clinical trial number: DRKS00028069, retrospectively registered).

### Injury prevention programme *Stop X*

The *Stop X* prevention programme was published by the German Knee Society in 2020. This prevention strategy consists of elements, such as active instruction on injury mechanisms and feedback how to avoid dangerous movement patterns. Additionally, the training programme incorporates running exercises, balance training, jump training and strength exercises [15]. The aim of this programme is to improve athletes’ neuromuscular control, thus reducing the risk of knee injuries. By targeting key muscle groups and enhancing coordination, the *Stop X* programme helps to build stability and strength around the knee joint. The *Stop X* prevention programme was incorporated into the 15–20-minute warm-up programme for every training session five to six times a week. The programme was integrated into the footballers’ daily training routines for two seasons. To ensure high compliance and quality of movement each team was supervised by an athletic coach who monitored the correct execution of the exercises according to the instructional protocol. Adherence was high, with an average completion rate of approximately over 85% of all scheduled training sessions.

### Jump tests

The initial measurements were conducted at the commencement of each respective season (July), with subsequent measurements taken halfway through the season (December/January) across the two-year study period. All participants underwent a standardised 15-minute warm-up on a bicycle ergometer prior to testing.

#### Equipment and setup

Vertical jump performance and kinematics were captured using a fully synchronised setup. Vertical ground reaction forces (vGRF) were recorded at a sampling frequency of 1000 Hz using Bertec force plates (Bertec, Columbus, OH, USA), connected via a Bertec AM6800 amplifier directly to the Noraxon myoRESEARCH® (MR3.20, Noraxon USA, Scottsdale, AZ, USA) software. 3D kinematics were captured using seven Ultium motion sensors (Noraxon USA, Scottsdale, AZ, USA) attached with Noraxon harnesses. The sensors were placed at the lower back (L5/S1), bilateral mid-thighs, upper tibiae, and dorsal feet. Data were collected at 200 Hz. A Logitech BRIO camera (Logitech International S.A., Apples, Switzerland) provided high-resolution video validation. The jumps were recorded with two lateral and one frontal camera at a height of 0.7 m (frontal), 0.6 m (left) and 0.64 m (right). All hardware was integrated and synchronised via Noraxon myoRESEARCH® (MR3.20) software.

#### Measurement protocol

Each session began with a reference pose using a Velamed mat (Velamed GmbH, Cologne, Germany) followed by a walking calibration to establish the kinematic reference. Participants performed four Countermovement Jumps (CMJ) and four Single-Leg Drop Jumps (SLDJ). For the CMJ, participants started from a standing position, performed a countermovement squat, and jumped vertically with maximal effort. For the SLDJ, participants dropped from a 20 cm box onto the force plate, followed by an immediate maximal vertical jump.

### Isokinetic tests

**Fig. 1:**
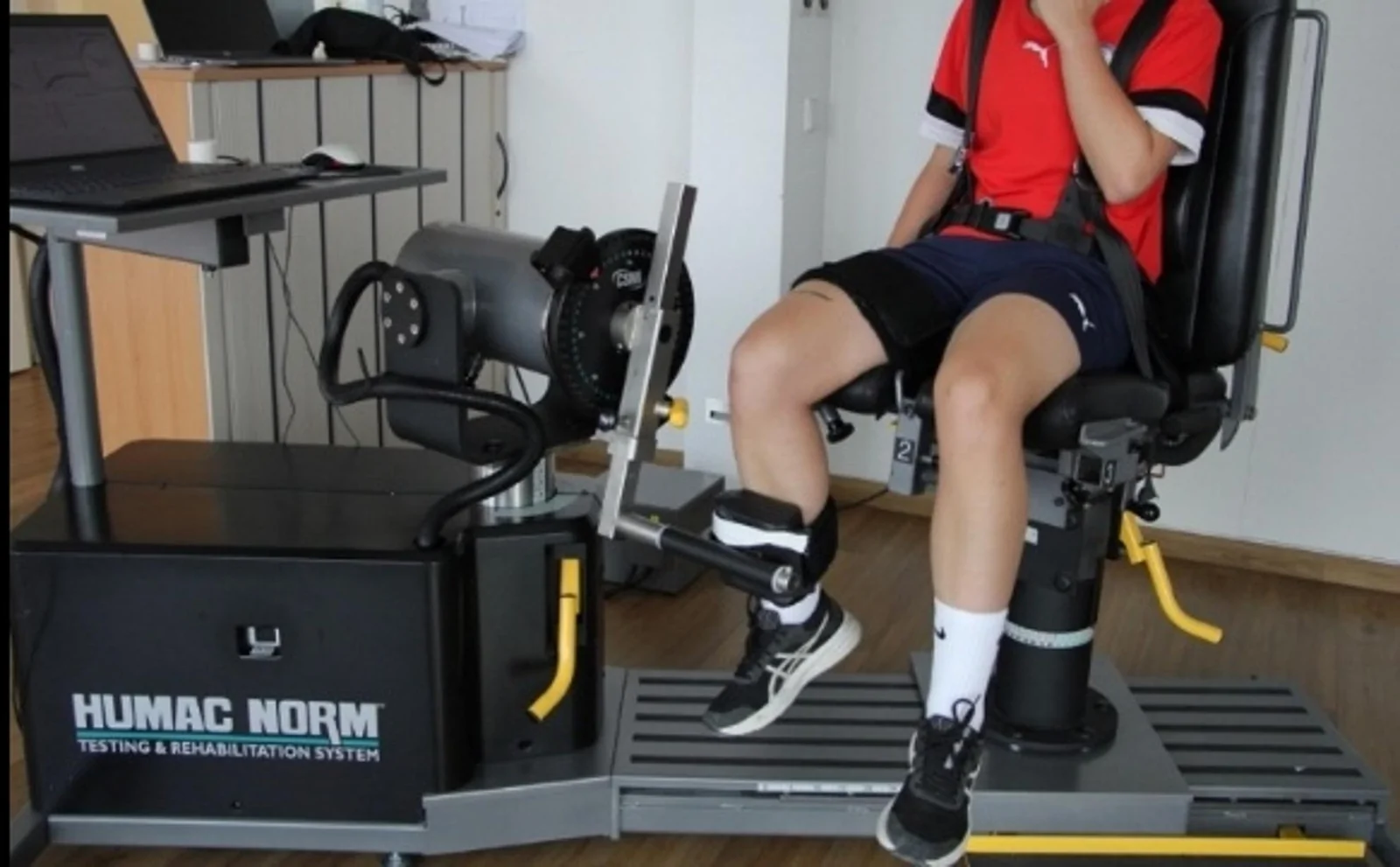
Setup of the isokinetic tests

The Humac Norm Isokinet (Computer Sports Medicine, Stoughton, MA, USA) was used to take the isokinetic measurements, with the testing and evaluation process conducted using the Humac 2015 software programme (Computer Sports Medicine, Stoughton, MA, USA). The test subjects were fastened to the chair via a shoulder/hip belt. The limb to be measured was secured to the chair by fastening the thigh to the chair, and the lower leg to the centre of the lever arm, ensuring that the knee was aligned with the fulcrum of the lever (see Fig. 1). After measuring the maximum passive extension and flexion, the endpoints of the movement were set, after which the lower leg’s passive weight was measured. The movement’s initial measurement was taken concentrically, with resistance applied during extension and flexion, at an angular velocity of 60°/s. After three test movements with reduced force, three repetitions at maximum force were measured. After a 45-second break, three test movements of eccentric flexion at 60°/s followed by three measurements of this movement at maximum force were taken. The tests’ mean values were then calculated.

### Evaluation/data analysis

### Jump tests

Data were analysed in Noraxon myoRESEARCH^®^ (MR3.20, Noraxon USA, Scottsdale, AZ, USA) using adapted protocols for CMJ and SLDJ. Jump height was calculated using the net impulse-momentum method, derived from the integration of the vGRF-time curve. The movement was segmented into three functional phases:

1. Eccentric Phase: From movement initiation (vGRF deviation > 20 N) to the point of zero velocity (lowest center of mass).
2. Concentric (Propulsive) Phase: From zero velocity to take-off (vGRF < 10 N). PPF was recorded as the maximum vertical net force (calculated as the total vGRF minus body weight) within this phase.
3. Landing Phase: From initial contact (vGRF > 10 N) to peak knee flexion.

For the CMJ, parameters included jump height, RSI and PPF. For the SLDJ, jump height and RSI were calculated. Knee abduction (valgus) values were quantified at the moment of initial contact (vGRF > 10 N) using the 3D sensor data and cross-validated with Kinovea^®^ (Version 0.9.3, open-source 2D motion analysis software, Kinovea Team). Following analysis, data were anonymised and transferred to Microsoft Excel (Microsoft Corporation, Redmond, WA), where the mean values of valid jumps were calculated for further statistical evaluation.

### Isokinetic tests

The best values of the isokinetic tests were derived from the measurement at 60°/s, and the H/Q ratios were calculated from both the concentric values and the flexion’s eccentric value.

### Statistical evaluation/data analysis

Statistical analysis was performed using SPSS version 29 (IBM Corporation, Armonk, NY, USA). The significance level was set at α = 0.05 (95% confidence interval). Normality of data distribution was assessed using the Shapiro-Wilk test, and homogeneity of variance was verified via Levene’s test. Descriptive statistics (minimum, maximum, mean, variance, and standard deviation) were calculated for all variables. To compare mean values between squads, one-way analysis of variance (ANOVA) or independent t-tests were used for normally distributed data. For non-normally distributed variables, the Kruskal-Wallis test or Mann-Whitney U test were applied. Each age group was also analysed separately to account for developmental differences.

Furthermore, an exploratory 1:4 matched-pair analysis was conducted to compare injured players with uninjured controls. Matching was based on Body-mass-index (BMI), height, weight, and injury history. Due to the specific demographic constraints of the cohort, tolerance ranges were established to ensure a 1:4 ratio: age (± 2 years), BMI (± 2 kg/m²), height (± 4 cm), and weight (± 2 kg). Differences within the matched pairs were analysed using independent t-tests or Mann-Whitney U tests, depending on the distribution.

Due to the specific nature of elite professional football, a formal a-priori power calculation was not performed. Instead, a convenience sample was used, comprising all available players from the participating professional and youth elite squads over the study period. While the final sample size of *n* = 147 is considered robust for detecting changes in neuromuscular performance markers (e.g., jump height and isokinetic strength), we acknowledge that the study is underpowered to establish a definitive causal link between the intervention and injury occurrence. This approach is consistent with the practical constraints of longitudinal studies in professional sports environments.

## Results

### Demographic and basic jump characteristics of participants

In this study, a total of 147 athletes, including 58 (39%) players on the U17-team, 34 players on the U20-team (23%), and 55 (37%) professional players, were enrolled in the seasons 22/23 and 23/24. The mean age of all participants was 19.03 years, while injuries occurred at a mean age of 19.35 years (for further demographic characteristics see Table 2). We observed significant differences in the force-dependent variables of the jump tests in the preseasons, while no significant differences in the valgus angles were found in the jump tests (see Table 2).

**Table 2:** Demographic characteristics and preseason jump variables of participants sorted by age group

Variable	U17 players	U20 players	Professional players	*p*-value
N	58	34	55	-
Age (yr)	15.1 ± 0.7	17.7 ± 0.7	23.8 ± 3.3	-
Height (cm)	165.1 ± 6.2	167.3 ± 5.6	170.1 ± 5.6	**< 0.001**
Weight (kg)	56.5 ± 6.3	59.8 ± 5.5	63.5 ± 6.6	**< 0.001**
BMI (kg/m2)	20.7 ± 1.78	21.5 ± 1.43	21.9 ± 1.46	**< 0.001**
CMJ peak propulsive force dominant (N)	626 ± 85	702 ± 94	759 ± 195	**< 0.001**
CMJ peak propulsive force non dominant (N)	615 ± 78	682.3 ± 77	749 ± 189	**< 0.001**
CMJ reactive strength index (m/s)	0.28 ± 0.06	0.36 ± 0.07	0.40 ± 0.07	**< 0.001**
CMJ knee abduction dominant (degree)	-0.74 ± 7.06	0.27 ± 4.78	0.01 ± 4.47	0.73
CMJ knee abduction non dominant (degree)	-1.25 ± 6.62	0.12 ± 4.63	0.06 ± 5.27	0.46
SLDJ knee abduction dominant (degree)	1.00 ± 8.27	0.59 ± 3.90	1.46 ± 4.33	0.71
SLDJ knee abduction non dominant (degree)	-0.82 ± 4.17	-0.13 ± 6.54	1.04 ± 6.07	0.77

Results are presented as numbers and mean ± SD. Statistically significant differences (*p* < 0.05) are indicated in bold

*Abbreviations*: *BMI *Body-Mass-Index, *CMJ *Countermovement jump, *SLDJ *Single leg drop jump. *U17/20* Under 17/20 years old players

### Comparison of inseason tests with preseason tests

#### Jump analysis

The inseason tests of seasons 2022/23 and 2023/24 of the CMJ and the SLDJ each demonstrated a considerably superior jump height compared to preseason tests (*p* < 0.001, *d* = 2.07, see Table 3). In terms of the directly force-dependent jump variables, the CMJ showed no significant differences between the preseason and inseason testing. However, in age-group subanalysis, there was a non-significant trend towards an improved CMJ reactive strength index (*p* = 0.08) and a significantly improved peak propulsive force (*p* = 0.04, *d* = 0.34) inseason for the U20 players. The SLDJ revealed an enhanced reactive strength index, with the U17 players showing substantial improvements (*p* = 0.01, *d* = 0.19 for the non-dominant leg and *p* < 0.001, *d* = 0.31 for the dominant leg) and in total (*p* = 0.05, *d* = 0.22 for the dominant leg, *p* = 0.08 for the non-dominant leg).

**Table 3:** Comparison of preseason tests with inseason tests overall

Variable		Mean ± SD (dom)	*p*-value	Mean ± SD (ndom)	*p*-value
CMJ jump height (cm)	Preseason	26.84 ± 4.65	**< 0.001**		
	Inseason	27.62 ± 4.93			
SLDJ jump height (cm)	Preseason	16.03 ± 4.54	***0.02***	14.94 ± 3.01	0.07
	Inseason	16.55 ± 3.47		15.58 ± 3.57	
CMJ reactive strength index (m/s)	Preseason	0.33 ± 0.08	0.17		
	Inseason	0.34 ± 0.08			
SLDJ reactive strength index (m/s)	Preseason	0.64 ± 0.23	*0.05*	0.59 ± 0.15	*0.08*
	Inseason	0.66 ± 0.16		0.61 ± 0.14	
H/Q-ratio 60° conventional	Preseason	0.58 ± 0.08	0.63	0.59 ± 0.08	0.08
	Inseason	0.58 ± 0.08		0.57 ± 0.09	
H/Q-ratio 60° functional	Preseason	0.79 ± 0.15	**< 0.01**	0.80 ± 0.14	**< 0.01**
	Inseason	0.75 ± 0.13		0.76 ± 0.14	

Neuromuscular and jump performance changes from pre-season to inseason (pooled intra-seasonal data from the 2022/23 and 2023/24 cycles). Values are presented as mean ± standard deviation. Statistically significant differences (*p* < 0.05) are indicated in bold. italic=Wilcoxon-Test, roman = *t*-test

*Abbreviations*: *dom *Dominant leg, *ndom* non-dominant leg, *CMJ *Countermovement jump, *SLDJ *Single leg drop jump

#### Isokinetic analysis

The isokinetic tests revealed a lower functional H/Q ratio at 60°/s for both legs (*p* < 0.01, *d* = 0.21 dominant and *d* = 0.20 non-dominant, see Table 3). In subgroup analysis, the U17s and professional players presented a significantly lower functional H/Q ratio inseason compared to preseason tests (e.g., non-dominant H/Q ratio of professional players in preseason 0.78 ± 0.14 vs. 0.71 ± 0.13, *p* = 0.05, *d* = 0.18). There was no significant difference in the conventional H/Q ratio of both legs in total. However, we observed a significant reduction in the conventional H/Q ratio of the professional players’ non-dominant legs in subgroup analysis (preseason 0.60 ± 0.07 vs. inseason 0.55 ± 0.11, *p* = 0.04, *d* = 0.19).

We analysed the initial contact coronal angles during the CMJ and SLDJ. In sum, the mean values of the cohort of players in this study during the preseason and inseason measurements revealed on average no strong deviations in knee abduction. A comparison of measurements from the two seasons showed a non-significant improvement in coronal angles during the programme’s implementation (see Table 4).

**Table 4:** Comparison of abduction values preseason jump tests with inseason jump tests overall

Jump test	Leg	Mean ± SD PS 23/24	Mean ± SD IS 22/23	Mean ± SD PS 23/24	Mean ± SD IS 23/24
Countermovement Jump (degree)	Left	-5.47 ± 6.15	1.25 ± 4.34	1.88 ± 2.98	1.22 ± 4.46
	Right	-3.43 ± 7.46	3.08 ± 6.71	2.42 ± 2.62	1.26 ± 3.24
Single leg drop jump (degree)	Left	1.53 ± 6.60	-0.51 ± 6.71	0.36 ± 4.48	0.99 ± 2.02
	Right	2.53 ± 6.98	3.67 ± 7.65	2.05 ± 2.86	1.90 ± 2.02

Values are presented as mean ± standard deviation

*Abbreviations*: *IS *Inseason, *PS *Preseason

### Comparison of the inseason injured players to the uninjured players in the preseason and inseason tests

We documented a total of 25 non-contact lower limb injuries (17% of participants) entailing a minimum two-week absence from training or competition during the study period (excluding muscle injuries). The dominant leg was affected in 18 of the 25 injuries (72%). The most common injury location was the ankle, accounting for 11 cases (44%). Injuries to the knee including ACL-ruptures and meniscal tears occurred in 7 players (28%): one of these was a re-rupture, and two players had already suffered ACL injuries on the contralateral side. When analysing the seasonal distribution across the two-year period, the highest frequency of injuries occurred within the calendar month of October, accounting for 20% of all recorded injuries. The average age of injured players was 19.22 years, compared to 19.18 years for uninjured players. We conducted 1:4 case-control matching between injured and uninjured players in terms of height, weight, BMI and injury history.

#### Jump analysis

The injured players revealed a non-significant trend toward higher jump heights in both preseason and inseason assessments compared to uninjured players (see Table 5). The peak propulsive force and reactive strength index demonstrated no statistically significant group differences. Initial contact coronal angles during CMJ showed a non-significant trend toward greater knee adduction (more negative angles) in injured players compared to uninjured peers. No significant group differences were detected in SLDJ landing angles of both legs.

**Table 5:** Comparison of inseason injured players with uninjured players overall

		Dominant leg				Non dominant leg			
Variable		Mean ± SD ps	*p*-value	Mean ± SD is	*p*-value	Mean ± SD ps	p-value	Mean ± SD is	*p*-value
CMJ jumpheight (cm)	No injury	26.63± 4.59	0.10	26.80± 4.55	0.07	-			
	Injury	28.50± 4.87		28.79± 5.75					
CMJ Peak propulsive force (N)	No injury	696± 147	0.44	694± 122	0.43	682 ± 140	0.67	684 ± 113	0.44
	Injury	716± 161		671± 146		697 ± 149		662 ± 145	
CMJ knee abduction (degree)	No injury	0.49 ± 5.62	*0.16*	2.24 ± 5.56	*0.19*	-0.22 ± 5.40	*0.12*	0.80 ± 4.64	*0.70*
	Injury	-1.47 ± 5.92		0.36 ± 5.03		-2.44 ± 6.92		0.32 ± 5.23	
SLDJ knee abduction (degree)	No injury	1.62 ± 5.96	*0.81*	2.13 ± 6.84	*0.88*	0.80 ± 5.17	*0.59*	0.22 ± 5.82	*0.99*
	Injury	1.84 ± 4.48		1.79 ± 3.25		-0.65 ± 5.74		-0.12 ± 4.24	
H/Q-ratio 60°conv.	No injury	0.59 ± 0.09	0.23	0.59 ± 0.08	**0.03**	0.58 ± 0.08	0.67	0.58 ± 0.08	0.45
	Injury	0.56 ± 0.07		0.54 ± 0.07		0.59 ± 0.07		0.56 ± 0.09	
H/Q-ratio 60° funct.	No injury	0.80 ± 0.15	0.43	0.75 ± 0.14	0.95	0.78 ± 0.13	0.11	0.74 ± 0.13	0.23
	Injury	0.77 ± 0.14		0.75 ± 0.12		0.84 ± 0.15		0.78 ± 0.14	

Values are presented as mean ± standard deviation. Statistically significant differences (*p* < 0.05) are indicated in bold. italic=Mann-Whitney-U-Test, roman = t-test

*Abbreviations*: *ps *preseason, *is *inseason, *CMJ *Countermovement Jump, *conv*. conventional, *funct*. functional. *SLDJ *Single leg drop jump

#### Isokinetic analysis

Isokinetic testing revealed that injured players presented a significantly lower conventional H/Q ratio in the dominant leg during the in-season assessment (see Table 5, *p* = 0.03, *d* = 0.19). No significant differences in the H/Q ratio—conventional or functional—were found in the non-dominant leg between injured and uninjured players.

### Evaluation by type of injury

An exploratory risk analysis was conducted to descriptively evaluate the biomechanical profiles of the injured players. Due to the low occurrence of injury events depending on the type of injury, the study is underpowered to draw firm conclusions regarding injury risk based on preseason and in-season measurements. Therefore, these descriptive findings should be considered hypothesis-generating. For the evaluation we conducted 1:4 case-control matching between injured and uninjured players in terms of height, weight, BMI and previous injuries.

#### Ankle injuries

Players who sustained ankle injuries tended to exhibit a varus leg axis during jump tests before their injury. However, this tendency failed to reach statistical significance when compared to uninjured players during the season. Similarly, no significant differences were observed in the H/Q ratio during isokinetic testing between players with ankle injuries and those without injuries (see Table 6).

**Table 6:** Comparison of preseason isokinetic and jump tests of matched inseason injured players with uninjured players

Variable	Mean ± SD ACL matched uninjured players	Mean ± SD ACL injured players	*p*-value	Mean ± SD knee matched uninjured players	Mean ± SD Knee injured players	*p*-value	Mean ± SD ankle matched uninjured players	Mean ± SD ankle injured players	*p*-value
Age (yr)	20.37± 4.19	21.14 ± 4.41	0.53	17.28 ± 3.29	17.71 ± 3.90	0.47	18.71 ± 4.28	19.55 ± 5.10	0.66
BMI (kg/m2)	22.12± 1.70	22.53 ± 1.69	0.64	21.35 ± 1.86	21.11 ± 2.26	0.58	21.09 ± 1.20	21.09 ± 1.11	0.98
CMJ knee abduction dominant (degree)	-3.57± 5.38	3.41 ± 5.38	*0.06*	0.60 ± 8.07	2.70 ± 5.22	*0.45*	0.42 ± 6.51	-1.58 ± 4.96	*0.15*
CMJ knee abduction non-dominant (degree)	-3.08± 5.28	3.23 ± 7.38	*0.09*	0.85 ± 5.18	1.61 ± 9.18	*0.34*	-1.08 ± 5.20	-2.31 ± 6.91	*0.30*
SLDJ right knee abduction (degree)	1.46± 5.89	3.20 ± 4.91	*0.20*	1.77 ± 6.23	4.30 ± 3.74	*0.14*	0.87 ± 4.72	-1.86 ± 2.39	*0.42*
SLDJ left knee abduction (degree)	-0.13± 3.28	1.16 ± 6.84	*0.29*	0.81 ± 2.89	0.10 ± 4.70	*0.68*	1.09 ± 5.80	-1.35 ± 5.49	*0.32*
H/Q-ratio 60° conv. dominant	0.59 ± 0.09	0.55 ± 0.06	0.10	0.59 ± 0.08	0.58 ± 0.04	0.30	0.60 ± 0.08	0.58 ± 0.10	0.57
H/Q-ratio 60° conv. non dominant	0.58 ± 0.09	0.53 ± 0.06	0.09	0.58 ± 0.08	0.59 ± 0.07	0.67	0.62 ± 0.06	0.61 ± 0.06	0.25
H/Q-ratio 60° funct. dominant	0.80 ± 0.16	0.78 ± 0.12	0.42	0.77 ± 0.17	0.76 ± 0.12	0.80	0.80 ± 0.14	0.76 ± 0.14	0.13
H/Q-ratio 60° funct. Non dominant	0.80 ± 0.14	0.73 ± 0.09	0.07	0.77 ± 0.17	0.79 ± 0.17	0.37	0.78 ± 0.13	0.81 ± 0.11	0.39

Values are presented as mean ± standard deviation. *p*-values represent significance levels for differences to matched controls. Abduction angles are measured in degree. italic=Mann-Whitney-U-Test, roman = *t*-test

*Abbreviations*: *BMI *Body-mass-index, *CMJ* Countermovement Jump, *SLDJ *Single leg drop jump, *Conv*. Conventional, *funct*. functional

#### Knee injuries

Players who sustained meniscal or collateral ligament injuries likewise showed no substantial deviations in their H/Q ratios compared to their uninjured counterparts. While jump testing, these players exhibited a non-significant trend toward increased knee abduction (valgus alignment) compared to uninjured players.

#### ACL-injuries

Matched ACL-injured players displayed higher—although non-significant—jump heights in both the SLDJ and CMJ. For example, CMJ jump height averaged 29.20 cm in ACL-injured players versus 26.51 cm in uninjured players (*p* = 0.07). Matched ACL-injured players demonstrated a quadriceps dominance, which was reflected in their lower H/Q ratio. Moreover, these injured players presented an increased dynamic knee valgus during jump tests (see Table 6).

## Discussion

The aim of this cohort study was to implement and evaluate the *Stop X* injury prevention programme in elite adolescent and professional female football players over two competitive seasons. The influence of the programme was assessed by applying isokinetic strength testing and jump analyses targeting surrogate markers and functional injury risk factors associated with non-contact lower limb injuries, particularly ACL injuries.

### Improvement of jump performance

Our results indicate significant improvements in jump performance, with both the CMJ and SLDJ showing increased jump heights during the inseason assessments compared to preseason. Additionally, there was an improvement in the SLDJ’s reactive strength index. These findings are consistent with research demonstrating an enhanced jump performance following the implementation of injury prevention programmes [16–18]. Strengthening and jump exercises should be an essential component of neuromuscular training programmes, as these lead to a significantly lower risk of injury compared to programmes lacking strengthening elements [19]. However, the protective effect of such strengthening is closely linked to how this strength is utilised to control joint kinematics during dynamic tasks. Specifically, suboptimal neuromuscular control (e.g., reduced activation of the gluteus medius) can lead to unfavourable leg alignment and thus to an additional increase in injury risk [20]. This mechanical instability is further exacerbated during high-intensity landings or in individuals with an elevated BMI, where the passive stabilisers of the knee must compensate for higher peak impact forces [21, 22]. The trend that subsequently injured players also displayed superior jump performance metrics may be linked to higher playing exposure, chronic workloads, or explosive positional demands (e.g., wingers or forwards) that simultaneously influence athletic capacity and cumulative injury risk. The integration of jumping and landing techniques focusing on quality rather than quantity within the prevention of knee injuries, particularly in women’s football, might be therefore of great importance [20–27].

### Dynamic knee valgus in jump tests

The name *Stop X* itself reflects its focus on addressing valgus knee positions—a known risk factor for ACL injuries [14, 15, 28, 29]. We detected a modest reduction in dynamic knee valgus in our cohort’s jump tests during the seasons when the *Stop X* prevention programme was being implemented (see Table 4). Similar improvements were documented in a study of 30 female basketball players using the *Stop X* programme in comparison to a standard warm-up [30]. Additionally, implementation of the *Stop X* programme has been associated with improvements in both static and dynamic balance in adolescent football players [31].

While we identified no significant difference in knee abduction angles when comparing the injured and uninjured cohort and specifically players with meniscus and ankle injuries, players who subsequently sustained an ACL injury exhibited significantly greater pre-injury valgus angles compared to uninjured players. However, due to the low number of non-contact ACL injuries these markers must be viewed as associated factors within this study population, rather than validated predictors. In a paper published by *Corban et al.*, initial coronal knee angle exceeding 2.96° has been associated with an increased ACL injury risk [29], which closely approximates the > 3° initial coronal abduction angles observed in our ACL-injured players in the jump tests. While these trends provide valuable insights for targeted neuromuscular training, their predictive value remains to be established in larger, prospective trials. Nevertheless, the clinical efficacy of neuromuscular prevention programmes is supported by broader evidence. Meta-analyses confirm that multi-component, exercise-based prevention programmes significantly reduce the risk of injury in female football, with reductions of 27% in total injuries and 45% in ACL injuries [9, 13]. Although bigger studies are needed, these results may emphasise the practical value of jump-based screening assessments as an accessible method to identify potential injury risk factors, particularly in adolescent female football players and resource-limited environments where isokinetic testing may not be feasible.

###  H/Q-ratio and muscular imbalances

Despite the strengthening components of the *Stop X* programme, we observed a slight decrease in the H/Q ratio during the in-season testing compared to preseason. This reduction may indicate a relative underemphasis of hamstring-specific training, despite the inclusion of Nordic hamstring exercises as a core component of the *Stop X* programme [14]. Studies have shown that the regular implementation of Nordic hamstring exercises leads to significant improvements in both functional and conventional H/Q ratios [32, 33] and has a substantial injury-preventive effect, especially regarding hamstring injuries in football players [34–36]. Most of these investigations, however, were conducted with male athletes exclusively. Notably, hamstring exercises are not necessarily suitable for all individuals. They are technically difficult to execute, particularly for women and those with limited practice. There is evidence that adolescents and women often have difficulty performing the Nordic hamstring exercise correctly, particularly at the angles at which the hamstrings are trained [37–40]. Therefore, it is conceivable that Nordic Curls exercises are not suitable for all women and adolescent athletes to ensure that the hamstrings are adequately trained. Alternatively, the frequency of Nordic hamstring curls within the *Stop X* protocol may have been too low to enable enough stimulus. However, our athletes participated in the *Stop X* prevention programme at least five times per week, and thus at a sufficient frequency [41]. Additional hamstring-specific strengthening exercises, i.e., the Romanian deadlift, may be added to ensure sufficient muscular engagement and a balanced H/Q ratio [42] especially considering that ACL injured players yielded a lower conventional H/Q ratio than those who remained injury-free. However, comparatively speaking: a recent meta-analysis suggests that the H/Q ratio may not be an independent risk factor for hamstring or ACL injuries [43, 44] and only one of several potential surrogate markers for non-contact lower limb injuries [44, 45]. Nonetheless, identifying and diminishing muscular imbalances remains essential in the context of preventing non-contact ACL and lower limb injury [44].

### Strength and limitations

The strengths of this prospective cohort study include the implementation of a neuromuscular prevention programme across two full seasons within three competitive women’s football teams of different age groups. By conducting the intervention under real-world training conditions, the study achieves high external validity and practical relevance for team sports. Furthermore, the focus on female athletes targets a high-risk population for non-contact ACL injuries. The use of standardised, well-established protocols for both isokinetic and jump assessments ensures measurement reliability and comparability across different time points.

Several limitations must be acknowledged. First, the absence of a randomised control group limits causal statements regarding the programme’s direct effectiveness; observed changes may also be influenced by the regular training load. Second, the study is underpowered to detect significant changes in clinical injury occurrence. With only seven recorded non-contact ACL injuries, the statistical evidence is insufficient to claim a direct reduction in injury risk. As previously estimated, approximately 120 players would need to participate in such a programme to prevent one ACL injury [46], highlighting the challenges of proving injury reduction in single-club cohorts. Consequently, the findings from this exploratory case-control matching serve to generate new hypotheses rather than provide definitive predictive parameters. Third, although assessments were standardised, repeated exposure to testing procedures may have introduced learning effects. Additionally, while jump and isokinetic tests are utilised as injury surrogate and functional risk factor markers, dynamic knee valgus measurements remain sensitive to methodological variability despite our 3D/2D cross-validation; thus, small angular differences should be interpreted with caution regarding their clinical relevance. Furthermore, they cannot fully replicate the unanticipated, multi-planar scenarios of real-game situations where injuries typically occur. Biomechanical laboratory data should therefore be considered alongside other multifactorial risks, such as psychological factors. For instance, a fear of reinjury has been shown to be a significant factor in athletes who suffer subsequent ACL injuries [47, 48]. Additionally, unmeasured confounders inherent to elite football, such as exact individual match/training exposure, playing position and seasonal workload may have influenced both longitudinal performance and injury outcomes. Consequently, these biomechanical tools identify potential risk associations rather than definitive predictors of injury.

### Implications

The findings of this study have several practical implications for strength and conditioning coaches and medical staff in women’s football. First, the observed improvements in jump performance indicate positive adaptations in neuromuscular capabilities during the tracking period. Second, the stability of dynamic knee valgus despite increased force production suggests that the programme supports safer movement patterns. However, the recorded decline in H/Q ratios during the competitive season indicates the necessity of refining the prevention programme, particularly for young women. Finally, while biomechanical screening can identify potential risk associations, it should be integrated into a multifactorial monitoring system. Future research should focus on randomised controlled trials with larger sample sizes to better assess the efficacy of prevention programmes and the validity of identified risk factors.

## Conclusion

Improvements in jump performance and propulsive forces were observed during the implementation of *Stop X* across professional and youth elite female football players. The observed performance gains are of particular clinical relevance, as they facilitate high compliance among coaching staff, a common barrier to the long-term implementation of prevention protocols. However, targeted adaptations to better address hamstring strength, as well as jumping and landing strategies, could further enhance its injury-preventive potential in women’s football. While large-scale randomised controlled trials are required to definitively assess the impact on injury occurrence, our findings support the continued implementation and refinement of structured neuromuscular training. These programmes remain essential in the effort to reduce the gender gap in lower limb injuries at both youth and professional levels of female football.

## Supplementary Information

Supplementary Material 1.

## Data Availability

The datasets used and/or analysed during the current study are available from the corresponding author on reasonable request.

## Abbreviations

ACL: Anterior cruciate ligament
BMI: Body-mass-index
CONV: Conventional
CMJ: Countermovement jump
DOM: Dominant
FUNCT: Functional
H/Q: Hamstrings-to-quadriceps
IS: Inseason
ND: Non dominant
PPF: Peak Propulsive Force PS preseason
RSI: Reactive Strength Index
SLDJ: Single-leg drop jump
U: Under
