# Supplementary material for: Preventing lower limb injuries in women’s football: Influence of the Stop X prevention programme on jump performance and isokinetics in professional and young elite-level female football – a prospective observational cohort study
Source: BMC Sports Sci Med Rehabil. 2026 Sep 9;18:418. doi: 10.1186/s13102-026-02064-2 (PMC13576237; doi:10.1186/s13102-026-02064-2)
Supplement: Supplementary file 1 [file 13102_2026_2064_MOESM1_ESM.pdf]

Supplementary Material S1: Standardised Injury Surveillance Form:

Team/Player's name	
Position	
Date of injury [DD/MM/YYYY]	
Date of Return to Competition [DD/MM/YYYY]	
Days of absence	
Reason for absence	☐ Acute injury ☐ Chronic injury ☐ Disease
Injury Location	☐Head ☐ Upper extremities ☐ Hip ☐ Thigh ☐ Knee ☐ Ankle ☐ Other: ____________________
Type of injury	
Diagnosis	
Occasion	☐ Training ☐ Match
Injury side	☐ Left ☐ Right
Mechanism of injury	☐ Non-contact ☐ Contact
Ground	☐ Natural grass ☐ Artificial turf
Brief description/additional information	

This form was used for weekly data collection by the medical staff (physiotherapists) throughout the 2023/24 season. Collected data were subsequently transferred into a standardised electronic database (Microsoft Excel) for further analysis and team-specific monitoring.
